# Herbal medicine on cancer-related fatigue of lung cancer survivors: Protocol for a systematic review

**DOI:** 10.1097/MD.0000000000018968

**Published:** 2020-01-31

**Authors:** Chan-Young Kwon, Boram Lee, Kwan-Il Kim, Beom-Joon Lee

**Affiliations:** a Department of Clinical Korean Medicine, Graduate School, Kyung Hee University, Seoul; b Clinical Medicine Division, Korea Institute of Oriental Medicine, Daejeon; c Department of Internal Medicine, Division of Allergy, Immune and Respiratory System, College of Korean Medicine, Kyunghee University, Seoul, Republic of Korea.

**Keywords:** fatigue, herbal medicine, lung neoplasm, systematic review

## Abstract

**Background:**

Lung cancer is one of the most common cancers worldwide, and approximately half of the patients with lung cancer receiving chemotherapy suffer from cancer-related fatigue (CRF). Herbal medicines (HMs) have been used in Oriental countries for centuries as tonics. Various beneficial effects of HM on fatigue and cancer have been reported. However, the effectiveness and safety of HM for CRF in lung cancer patients have not been synthesized. The purpose of this systematic review is to evaluate the effectiveness and safety of HM for CRF in patients with lung cancer, regardless of their cancer type or stage.

**Methods and analysis:**

A comprehensive search will be conducted in 12 electronic medical databases including 5 English-language databases (Medline via PubMed, EMBASE via Elsevier, the Cochrane Central Register of Controlled Trials [CENTRAL], the Allied and Complementary Medicine Database [AMED] via EBSCO, and the Cumulative Index to Nursing and Allied Health Literature [CINAHL] via EBSCO), 4 Korean-language databases (Oriental Medicine Advanced Searching Integrated System [OASIS], Koreanstudies Information Service System [KISS], Research Information Service System [RISS], and Korea Citation Index [KCI]), 2 Chinese-language databases (China National Knowledge Infrastructure [CNKI] and Wanfang Data), and 1 Japanese-language database (CiNii). Only randomized controlled trials (RCTs) and quasi-RCTs on HM for CRF will be allowed. The severity of fatigue assessed using a validated tool will be considered as theprimary outcome. The secondary outcomes will include the patients’ quality of life, activities of daily life, incidence of adverse events, and total effective rate. Two independent researchers will perform the study selection, data extraction, and quality assessment. RevMan version 5.3 will be used for data synthesis. The methodological quality of the included RCTs will be assessed using the Cochrane Collaboration's risk of bias tool. In the meta-analysis, for dichotomous data and continuous data, risk ratio and mean difference, respectively, will be estimated with their 95% confidence intervals. According to the heterogeneity, either a fixed-effects or a random-effects model will be used.

**Ethics and dissemination:**

Ethical approval is not required because individual patient data are not included. The findings of this systematic review will be disseminated through a peer-reviewed publication or conference presentation.

**PROSPERO registration number:**

CRD42019141660.

## 1 Introduction

Lung cancer is one of the most common cancers worldwide, and 1.8 million new cases emerge annually.^[1]^ Treatment for lung cancer differs depending on the progression or the presence of metastasis, but surgical resection, molecular targeted therapies, combination chemotherapy, and stereotactic body radiation are considered as the main modes of treatment.^[2]^ Despite these treatments, fatigue in cancer patients, the so-called cancer-related fatigue (CRF), is a stubborn and distressing symptom that significantly reduces the quality of life (QOL) of the affected patients.^[3,4]^ The underlying pathophysiology of CRF has not been clearly elucidated, but may be due to the outcome of cancer itself and/or the side effect of chemotherapy and radiation therapy.^[5]^ According to an epidemiologic study, moderate levels of CRF are present in half of the patients with lung cancer who received chemotherapy, and persists for several months; radiation therapy may also affect the severity of CRF.^[6]^ In addition, insomnia, dyspnea, and cough in patients with lung cancer who received chemotherapy may also affect the CRF in these patients.^[7]^ Physical activity and diet are generally recommended to address this problem. However, apart from these lifestyle interventions, clinical evidence of treatment options for CRF in the clinical setting is limited.^[8,9]^

East Asian traditional medicine (EATM) modalities including herbal medicine (HM), acupuncture, and moxibustion have been widely used for health promotion, especially in the East Asian countries. Clinical evidences of the treatment benefits on outcome of the patients with fatigue and cancer have been accumulated.^[10–13]^ In particular, HM has been reported to have various beneficial effects on lung cancer as an effective adjuvant and maintenance therapy strategy^[14]^ in improving the survival rate^[15]^ and QOL,^[16]^ and promoting physiological improvement.^[17]^ It is important to identify the role of EATMs, a key element of complementary and integrative medicine (CIM), in situations where management based on CIM is important in the care of lung cancer.^[18]^ However, the impacts of HM on CRF in lung cancer patients have not yet been thoroughly reviewed.

The purpose of this systematic review is to evaluate the effectiveness and safety of HM for CRF in patients with lung cancer, regardless of the type or stage. The results of this review will help establish an integrated treatment strategy for CRF in lung cancer patients and enable the identification of effective evidence-based interventions that may improve the QOL of the patients.

## 2 Methods

### 2.1 Study registration

The protocol of this systematic review is registered in the International Prospective Register of Systematic Reviews, PROSPERO (registration number: CRD42019141660). If protocol amendments occur, the dates, changes, and rationales will be tracked in PROSPERO. This protocol was reported in accordance with the Preferred Reporting Items for Systematic Review and Meta-Analysis Protocols (PRISMA-P) 2015 statement.^[19]^

### 2.2 Data sources and search strategy

A comprehensive search will be conducted on September 2019, in 12 electronic medical databases, including 5 English-language databases (Medline via PubMed, EMBASE via Elsevier, the Cochrane Central Register of Controlled Trials [CENTRAL], the Allied and Complementary Medicine Database [AMED] via EBSCO, and the Cumulative Index to Nursing and Allied Health Literature [CINAHL] via EBSCO), 4 Korean-language databases (Oriental Medicine Advanced Searching Integrated System [OASIS], Korean Studies Information Service System [KISS], Research Information Service System [RISS], and Korea Citation Index [KCI]), two Chinese-language databases (China National Knowledge Infrastructure [CNKI], and Wanfang Data), and 1 Japanese-language database (CiNii). We will review the reference lists of relevant articles and manually search on Google Scholar to identify additional trials. Table 1 shows the search strategy in Medline via PubMed.

**Table 1:**
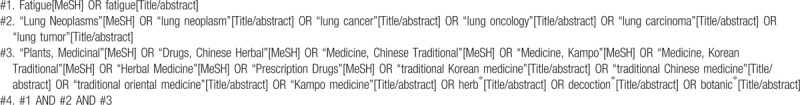
Search strategies for the Medline via PubMed.

### 2.3 Inclusion criteria

#### 2.3.1 Types of studies

Only randomized controlled trials (RCTs) and quasi-RCT study design using a quasi-random method such as alternate allocation or allocation by birthdate will be allowed.

#### 2.3.2 Types of participants

Regardless of its stage or severity, CRF patients with any type of lung cancer including small cell lung cancers and non-small cell lung cancers will be allowed. There were no limitations in the types of diagnostic criteria used to screen patients for CRF; however, studies without describing diagnostic criteria or tools were excluded. We will exclude the trials that included participants suffering from drug allergies or other serious illnesses such as other cancers, liver disease, or kidney disease.

#### 2.3.3 Types of interventions

Only oral HMs prescribed on the basis of the EATMs theory including traditional Chinese medicine, Kampo medicine, or traditional Korean medicine, will be allowed under experimental interventions. There is no restriction on the formulation of HM. However, we will exclude studies that do not list the composition of the HM used, except for patented drugs.

For control intervention, placebo, no treatment, or conventional medical treatments will be allowed. We will include studies involving HM combined with other therapies as experimental interventions if the other therapies are used equally in both the experimental and control groups. However, studies comparing different types of oral HM will be excluded.

#### 2.3.4 Types of outcome measures

The primary outcome measures were fatigue measured by the Brief Fatigue Inventory,^[20]^ the Profile of Mood States-Fatigue,^[21]^ the Fatigue Functional Assessment of Cancer Therapy-Fatigue subscale,^[22]^ numeral rating scale, and visual analogue scale.

The secondary outcome measures were as follows:

1. QOL measured by the Functional Assessment of Cancer Therapy-Lung Cancer Subscale^[23]^ and the European Organization for Research and Treatment of Cancer (EORTC) Quality of Life Questionnaire Core 30 (QLQ-C30) QOL measure.^[24]^
2. Activity of daily life-specific outcome such as the Karnofsky Performance Status Scale.^[25]^
3. The incidence of adverse events.
4. Total effective rate.

### 2.4 Study selection

**Figure 1:**
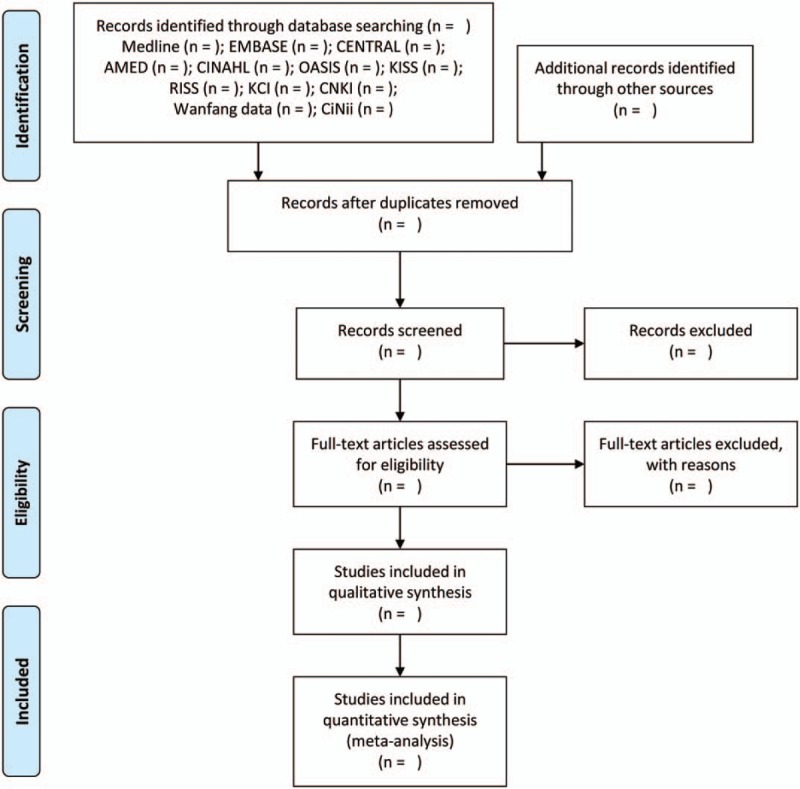
A PRISMA flow diagram of the literature screening and selection processes. AMED = Allied and Complementary Medicine Database, CENTRAL = Cochrane Central Register of Controlled Trials, CINAHL = Cumulative Index to Nursing and Allied Health Literature, CNKI = China National Knowledge Infrastructure, KCI = Korea Citation Index, KISS = Korean Studies Information Service System, OASIS = Oriental Medicine Advanced Searching Integrated System, RISS = Research Information Service System.

After performing database searches and removing duplications, the titles and abstracts of the searched studies will be screened for first inclusion. Then, the full texts of the potentially relevant articles will be evaluated for final inclusion. The entire study selection process will be performed by 2 authors (C-YK and BL) independently. Any disagreement between the 2 authors will be resolved through discussion with other researchers (K-I Kim). EndNote X8 (Clarivate Analytics, PA) will be used to manage quotations from included articles. The selection process and reasons for exclusions will be recorded in accordance with the PRISMA flow diagram (Fig. 1).

### 2.5 Data extraction

From the included studies, the following items will be extracted: study characteristics (first author, publication year, and country); approval from the institutional review board; informed consent; sample size and number of dropouts; diagnostic criteria; details about the participants, interventions, and comparisons; duration of the intervention and follow-up; outcome measures; outcomes; and adverse events. The data extraction process will be performed by 2 authors (C-YK and BL) independently. Any disagreement between the 2 authors will be resolved through discussion with other researchers (K-IK). Excel 2016 (Microsoft, Redmond, WA) and Dropbox (Dropbox, Inc., CA) folders will be used to perform the data extraction process, and to share the extracted data, respectively. We will contact the corresponding authors of the included studies via E-mail if the data are insufficient or ambiguous.

### 2.6 Quality assessment

The aim of this study was to assess the risk of bias of the included RCTs, the Cochrane Collaboration's risk of bias tool will be used. In this tool, 7 domains including random sequence generation, allocation concealment, blinding of participants, personnel, and outcome assessors, completeness of outcome data, selective reporting, and other biases are assessed as “low risk,” “unclear risk,” or “high risk.”^[26]^ The quality assessment process will be performed by 2 authors (C-YK and BL) independently. Any disagreement between the 2 authors will be resolved through discussion with other researchers (K-IK).

### 2.7 Data synthesis and analysis

Descriptive analyses will be conducted for all the included studies. Across the studies using homogeneous types of interventions, comparisons, and outcome measures, we will perform a meta-analysis using the Review Manager version 5.3 software (Cochrane, London, UK). We will pool the continuous data using the mean difference or standardized mean difference with 95% confidence intervals (CIs) and the dichotomous data using the risk ratio with 95% CIs. Heterogeneity will be assessed using both the *χ*^2^ test and the *I*^2^ statistic. It will be considered that *I*^2^ values ≥50% and ≥75% indicate substantial and considerable heterogeneity, respectively. In the meta-analyses, when the heterogeneity is significant (an *I*^2^ value ≥50%) we will use a random-effects model, and otherwise a fixed-effects model. A fixed-effects model will also be used when the number of studies included in the meta-analysis is <5.^[27,28]^

#### 2.7.1 Subgroup analysis

If the collected data are sufficient, we will perform a subgroup analysis according to the following criteria: types of lung cancer, presence or absence of pattern identification, and types of conventional medicine.

#### 2.7.2 Sensitivity analysis

To identify the robustness of the results of the meta-analysis, we will perform sensitivity analyses by excluding studies with high risks of bias and outliers that are numerically distant from the rest of the data.

#### 2.7.3 Assessment of reporting biases

If >10 RCTs are included in the meta-analysis, reporting biases including publication bias will be assessed using funnel plots.

### 2.8 Ethics and dissemination

Ethical approval is not required because this protocol is for a systematic review and not a clinical study. The results will be disseminated by the publication of a manuscript in a peer-reviewed journal or presentation at a relevant conference.

## 3 Discussion

Lung cancer is the most common cancer responsible for causing death worldwide,^[1]^ and many individuals with lung cancer suffer from CRF.^[6]^ As the CIM approach becomes important in cancer care, a comprehensive strategy including lifestyle intervention, in addition to conventional medicine treatments, is gaining importance.^[18]^ In particular, diet and physical activity are considered important approaches to improve CRF.^[8,9]^ However, more treatment strategies are needed to address this problem.

EATMs modalities such as acupuncture, moxibustion, and HM have long been used for health promotion, fatigue, and cancer,^[10–13]^ especially in the East Asian countries. Although the exact use of HM for CRF has rarely been studied, according to a national cross-sectional survey conducted in Japan, 64.3% of the physicians working in the palliative care unit use HM for cancer-related problems, and 32.0% of these comprised of CRF.^[29]^ Moreover, HM has been reported to have various beneficial effects to improve survival rate, and QOL of patients with lung cancer.^[15,16]^

It is important to establish evidences of the use of the EATMs modality in the holistic management of lung cancer patients.^[18]^ However, as far as we know, there has been no attempt to systematically synthesize clinical evidences supporting the use of HM for CRF in lung cancer patients. We believe that our findings will help patients, clinicians, and decision-makers establish effective comprehensive management plans for patients with lung cancer.

## Author contributions

The study was conceptualized by KIK and BJL. The search strategy was developed by CYK and BL. The protocol was drafted by CYK and BL. CYK, BL, KIK and BJL revised the manuscript. CYK submitted the manuscript for publication. All authors have read and approved the final manuscript.

Chan-Young Kwon orcid: 0000-0003-0068-9904.
